# Predicting Oral Food Challenge Outcomes in Cow’s Milk Protein Allergy: The Crucial Role of Bos d 8 and Component-Resolved Diagnostics: A Narrative Review

**DOI:** 10.3390/ijms27083643

**Published:** 2026-04-19

**Authors:** Weronika Balas, Aleksandra Tylewicz, Weronika Gromek, Weronika Sobota, Adam Sybilski, Emilia Majsiak

**Affiliations:** 1 Clinical Department of Paediatrics and Allergology, National Medical Institute of the Ministry of the Interior and Administration, 02-507 Warsaw, Poland; weronika.balas@pimmswia.gov.pl (W.B.); weronika.sobota@pimmswia.gov.pl (W.S.); 2 Polish-Ukrainian Foundation of Medicine Development, Nałęczowska 14, 20-701 Lublin, Poland; weronikaa.gromek@gmail.com; 3 Department of Paediatrics, Centre of Postgraduate Medical Education, 01-813 Warsaw, Poland; 4 Department of Health Promotion, Faculty Health of Sciences, Medical University of Lublin, Staszica 4/6, 20-081 Lublin, Poland

**Keywords:** cow’s milk allergy (CMPA), component-resolved diagnostics (CRD), IgE-mediated food allergies, oral food challenge, casein

## Abstract

Approximately 1–2% of infants have cow’s milk protein allergy (CMPA). From a clinical perspective, diagnosing CMPA using the oral food challenge (OFC) is high risk, necessitating safer alternatives. One possible alternative is component-resolved diagnostics (CRD). This narrative review examines specific IgE (sIgE) thresholds for cow’s milk protein in predicting outcomes of OFCs in European children. Eligible studies focusing on CRD in European pediatric populations were identified through PubMed and Scopus databases. Our findings highlight the crucial role of Bos d 8 (casein) in the diagnostic process. Among the analyzed milk components, Bos d 8 appeared to be a promising marker for predicting positive OFC outcomes in several cohorts. However, due to significant population heterogeneity, conflicting findings exist, with some studies indicating that no single molecular component is consistently superior to whole cow’s milk specific IgE. While other molecules, such as Bos d 6 and lactoferrin, showed limited diagnostic utility, specific IgE to Bos d 8 demonstrated the highest clinical value. Although the double-blind, placebo-controlled food challenge (DBPCFC) remains the gold standard for CMPA diagnosis, the use of Bos d 8 in CRD is a key step toward risk stratification and may help reduce the need for high-risk OFCs in selected patients.

## 1. Introduction

The most common allergy occurring in infancy is cow’s milk protein allergy (CMPA), affecting approximately 1–2% of infants [1]. The gold standard for diagnosing and monitoring or confirming resolution remains an oral food challenge (OFC) [2]. It is a procedure involving the administration of increasing amounts of a solution containing the suspected allergen throughout the day while monitoring the patient’s condition [3].

Each patient has an individualized plan for an OFC, selected based on the patient’s history and physical examination [1,4]. If the patient has a previous history of anaphylaxis, an OFC can be performed only if laboratory tests and medical history do not identify the food allergen or if there is a possibility of clinical tolerance [4]. After an OFC, the observation period should range from 2 to 6 h, depending on the patient’s response. If a patient develops any symptoms during the OFC, the elimination diet should be continued. However, if the patient does not experience any symptoms, milk can be reintroduced in a similar manner to the amounts used in OFC, using a milk ladder [4].

High levels of sIgE are associated with an increased likelihood of a severe reaction after consumption. The utility of sIgE levels in diagnosing and predicting the symptoms of cow’s milk allergy has been well-established. The proposed cut-off values for sIgE to whole milk for diagnosing CMPA without the need for OFC vary, but their utility remains disputed [5,6,7,8]. More comprehensive component-resolved diagnostics (CRD) are increasingly used in clinical practice to enhance the diagnostic process, which aligns the focus of this study [9]. In recent years, there has been increased interest in innovative methods of diagnosing CMPA. Numerous studies are emerging on the diagnosis of CMPA in children with suspected CMPA. These studies correlate sIgE levels with the results of an oral challenge test, as well as evaluate the ability of serial sIgE measurements to distinguish children with persistent CMPA from those who develop tolerance [10]. The identified allergens of CMP include Bos d 4 (α-lactalbumin), Bos d 5 (β-lactoglobulin), Bos d 6 (bovine serum albumin), Bos d 7 (immunoglobulins), Bos d 8 (caseins), and Bos d lactoferrin (lactoferrin). In diagnostics, the relevant CMP are casein (Bos d 8) and whey proteins (Bos d 4, Bos d 5). They all belong to the major CMP allergens and are commonly used to determine sIgE levels when diagnosing CMPA.

The aim of this study is to review manuscripts describing the impact of component-resolved diagnostics on OFCs in the European pediatric population. Additionally, the study compiles researchers’ proposed values of specific IgE for cow’s milk proteins, indicating the severity of the reaction or the possibility of acquiring tolerance to cow’s milk proteins. To reduce heterogeneity and improve comparability of diagnostic thresholds, this review was restricted to studies conducted in European pediatric populations, where clinical practice and diagnostic approaches to cow’s milk protein allergy are relatively consistent.

## 2. Results

To identify cut-off values to any sIgE to cow’s milk protein, 10 publications were included in this analysis: 5 original retrospective studies and 5 original prospective studies. The 10 papers were identified through Scopus and PubMed, available in full text, after excluding articles that were off topic or did not meet the inclusion criteria. In our analysis, we focused on evaluating the diagnostic effectiveness of sIgE to Bos d 4, 5, 6, and 8. Therefore, parameters such as cut-off value, negative predictive value (NPV), positive predictive value (PPV), sensitivity, specificity, and Area Under the Curve (AUC) for clinically significant cow’s milk molecules were taken into consideration. The key characteristics of the selected publications, including the specific OFC protocols utilized (e.g., open, the double-blind, placebo-controlled food challenge (DBPCFC), or mixed), are summarized in Table 1. In total, the cut-off points of sIgE values used to recommend whether to proceed with an OFC were analyzed in 706 children with CMPA. The applicability of component-resolved diagnostics in CMPA, including cut-offs, NPV, PPV, AUC, sensitivity, and specificity, as reported in the included studies, is presented in Table 2, Table 3, Table 4, Table 5 and Table 6.

### 2.1. Bos d 4

Among all analyzed papers, the Ayats-Vidal et al. study showed the highest AUC value (0.908) for α-lactalbumin, achieved with an sIgE threshold of 2.25 kU_A_/L. This retrospective article [15] involved 138 Spanish pediatric patients (mean age 4 years, range 0.75 to 15 years), 39 of whom were diagnosed with CMPA (Table 2) [15]. In another study by García-Ara et al. [7], the threshold value was set at 0.35 kU_A_/L. This value showed relatively high specificity (0.84), but only moderate sensitivity (0.55). PPV and NPV were relatively balanced, at approximately 0.74 and 0.7 [7]. However, in the largest patient group studied by Nieminen et al. [17] even with a threshold of 6.25 kU_A_/L, one of the highest sensitivities (0.92), the specificity remained low at 0.32 [12]. D’Urbano et al. [13] presented an AUC of 0.77 for a cut-off value of 34.27 kU_A_/L, with a PPV of 1.0, based on 32 CMPA patients [13].

The Ayats-Vidal study demonstrated high diagnostic reliability for CMPA, with the cut-off set on 2.25 kU_A_/L [15]. This conclusion is based on its high AUC (0.908) and a balanced sensitivity (0.67) and specificity (0.91). Studies with different age ranges (Nieminen et al., D’Urbano et al.) show more variable results, which may be influenced by factors like persistent CMPA or tolerance development [13,17].

**Figure 1:**
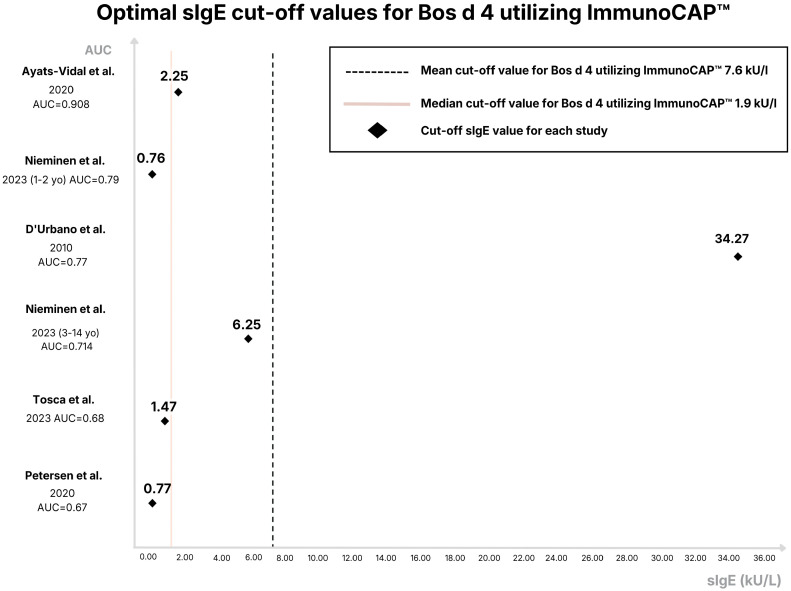
Distribution of sIgE cut-off values for Bos d 4 measured using ImmunoCAP. Across the analyzed studies, the mean cut-off value was 7.6 kU_A_/L (median: 1.9 kU_A_/L), and the reported Area Under the Curve (AUC) values ranged from 0.67 to 0.908 [10,13,15,17,18].

The cut-off values for specific IgE (sIgE) to Bos d 4 demonstrated significant variability. Reported values ranged from 0 to 34.27 kU_A_/L. The threshold values also varied between 0.35 and 34.27 kU_A_/L for ImmunoCAP testing. The range for ISAC testing was from 0 to 2.27 kU_A_/L. The sensitivity of sIgE measurements for α-lactalbumin was also highly variable. For ImmunoCAP testing, values ranged from 32% to 67% and for ISAC testing from 50% to 56%. Specificity similarly showed a broad range, with values reported between 71% and 93% for ImmunoCAP, and between 88% and 93% for ISAC. Notably, the levels of sIgE to Bos d 4 exhibited a relatively high PPV. For ImmunoCAP testing from 0.74 to 0.97, and for ISAC testing from 0.875 to 1. Furthermore, the NPV for α-lactalbumin sIgE was also reported to be reasonably high. ImmunoCAP values ranged from 0.67 to 0.908, and ISAC values approximated 0.7. The optimal cut-off values for sIgE to Bos d 4 using ImmunoCAP are presented in Figure 1. The mean cut-off value to Bos d 4 using ImmunoCAP was 7.6 kU_A_/L, and the median was 1.9 kU_A_/L with an AUC value of 0.67–0.908.

### 2.2. Bos d 5

**Figure 2:**
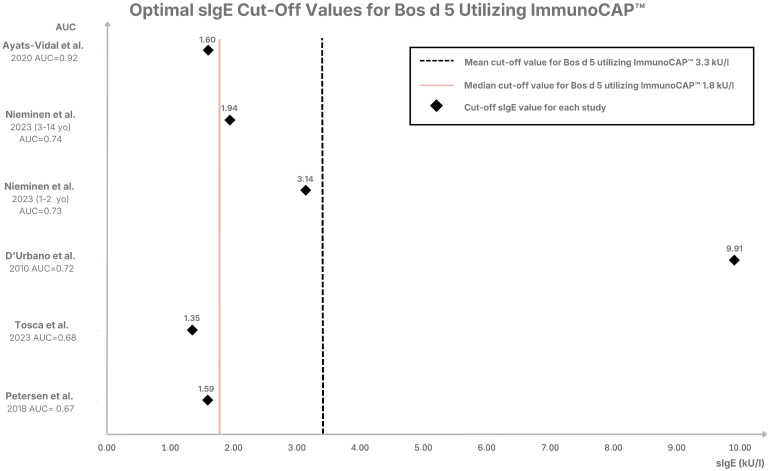
Distribution of sIgE cut–off values for Bos d 5 measured using ImmunoCAP. Across the analyzed studies, the mean cut–off value was 3.3 kU_A_/L (median: 1.8 kU_A_/L), and the reported Area Under the Curve (AUC) values ranged from 0.60 to 0.92 [10,13,15,17,18].

The cut-off thresholds for Bos d 5 differ significantly among reviewed studies on CMPA, highlighting the challenges in standardizing diagnostic criteria. Among the included studies, Ayats-Vidal et al. [15], a retrospective analysis of 138 Spanish children, achieved the highest diagnostic accuracy with a cut-off of 1.6 kU_A_/L. This study reports an excellent AUC of 0.92, high sensitivity (0.82), and specificity (0.91) [15]. In contrast, Nieminen et al. [17], a prospective Finnish study with the largest sample of 130 children, used a higher cut-off (1.94 kU_A_/L), which improved specificity (0.92) but reduced sensitivity (0.38). It resulted in a slightly lower AUC (0.74) [12]. Alessandri et al. [14] compared ISAC and ImmunoCAP methods in 75 Italian children and demonstrated greater effectiveness of ImmunoCAP. In the Italian study, sensitivity (0.5) and specificity (0.9) were balanced at a low cut-off of 1.02 kU_A_/L [14]. Ott et al. [11], using the lowest cut-off (0.1 kU_A_/L), achieved high specificity (0.95) but had the weakest sensitivity (0.24), with a modest AUC of 0.6 [11]. Neves et al. [16], a retrospective Portuguese study of 105 children, proposed a similar threshold of 1.7 kU_A_/L, achieving perfect PPV (1.0) and high specificity (0.95) but moderate sensitivity (0.58) [16]. Petersen et al. [10], a retrospective Danish study of 78 children, identified a higher cut-off of 1.59 kU_A_/L for Bos d 5 with modest sensitivity (0.44) and specificity (0.84), yielding an AUC of 0.67 [10]. Tosca et al. [18], an Italian retrospective analysis of 72 children, proposed a lower cut-off of 1.35 kU_A_/L, resulting in balanced sensitivity (0.59) and specificity (0.68) with an AUC of 0.68. Ayats-Vidal’s study is distinguished by the relatively balanced diagnostic performance [15]. The optimal cut-off values for sIgE to Bos d 5 in the analyzed studies using ImmunoCAP are presented in Figure 2. The mean cut-off value to Bos d 5 using ImmunoCAP was 3.3 kU_A_/L, and the median was 1.8 kU_A_/L, with an AUC value of 0.6–0.92.

In the screened studies, the cut-off values based on sIgE for Bos d 5 varied widely. They ranged from 0 to 9.91 kU_A_/L (for ImmunoCAP, 0.35 to 9.91 kU_A_/L; for ISAC, 0 to 0.1 ISU). The sensitivity for ImmunoCAP testing ranged from 30% to 82%, whereas for ISAC testing, it ranged from 24% to 40%. In ImmunoCAP testing, specificity values varied between 68% and 95%, while for ISAC testing, they were consistently high, ranging from 94% to 95%. The measurement of sIgE to Bos d 5 demonstrated a relatively high PPV, for both tests. For ImmunoCAP, PPV ranged from 0.62 to 0.95 and for ISAC, it ranged from 0.83 to 0.95. In contrast, the NPV for Bos d 5 sIgE was lower, ranging from 0.22 to 0.71 for ImmunoCAP and from 0.33 to 0.56 for ISAC.

### 2.3. Bos d 6

Bos d 6 cut-offs were assessed in two prospective studies. An Italian study using ImmunoCAP showed the highest diagnostic accuracy. This study demonstrated an AUC of 0.72 at 9.91 kU_A_/L, achieving perfect PPV (1.0) but moderate NPV (0.50). In the same study, the ISAC method had a lower AUC (0.59) and NPV (0.46) but retained a PPV of 1.0 [13]. Nieminen et al. proposed a much lower cut-off (0.49 kU_A_/L) [17]. This method presented high specificity but low sensitivity (6%) and a poor AUC (0.413), limiting its diagnostic utility.

The cut-off values for sIgE to Bos d 6 exhibited considerable variability across the studies analyzed, ranging from 0.26 to 44.32 kU_A_/L. The highest value was observed for ISAC (44.32 kU_A_/L) and for ImmunoCAP, the range was 0.26 to 9.91 kU_A_/L. In the Nieminen study, the 3–14-year-old group had a higher cut-off value (0.49 kU_A_/L) compared to the 1–2-year-old group (0.26 kU_A_/L) in the ImmunoCAP. The sensitivity for ImmunoCAP testing ranged from 6% to 8%. Unfortunately, no data were available for ISAC testing. Specificity values for ImmunoCAP testing varied between 92% and 93%, while for ISAC testing, data were also unavailable. The measurement of sIgE to Bos d 6 demonstrated a relatively high PPV. It ranged from 0.5 to 1 for ImmunoCAP and 1 for ISAC. sIgE for Bos d 6 showed the NPV ranging from 0.18 to 0.5 for ImmunoCAP and 0.46 for ISAC, which is lower than the PPV.

### 2.4. Bos d 8

**Figure 3:**
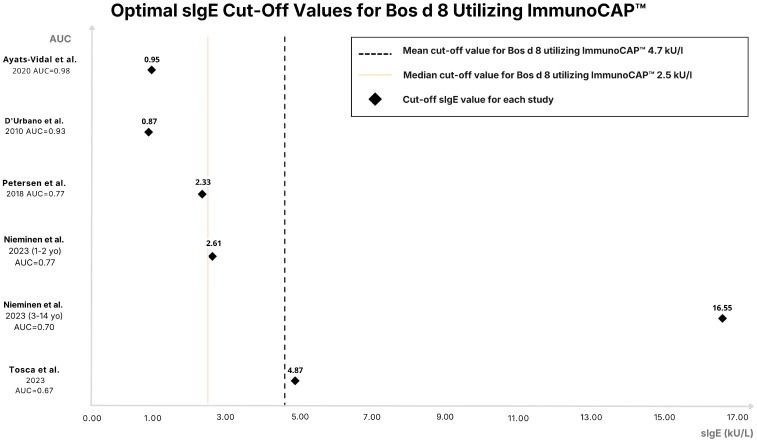
Distribution of sIgE cut–off values for Bos d 8 measured using ImmunoCAP. Across the analyzed studies, the mean cut–off value was 4.7 kU_A_/L (median: 2.5 kU_A_/L), and the reported Area Under the Curve (AUC) values ranged from 0.67 to 0.976 [10,13,15,17,18].

The cut-off points for sIgE for Bos d 8, which is a mixture of these casein fractions, vary significantly across reviewed studies. This diagnostic performance clearly reflects differences in population characteristics and methodologies. The Spanish study by Ayats-Vidal et al. [15] demonstrated a high diagnostic accuracy with a cut-off of 0.95 kU_A_/L. This method achieved a high AUC of 0.976 and well-balanced sensitivity (0.89) and specificity (0.91) [15]. Similarly to the Ayats-Vidal et al. study, D’Urbano et al. [13] also showed a high AUC (0.93) for ImmunoCAP with a cut-off of 0.78 kU_A_/L. Furthermore, Italian researchers investigated specific casein fractions. This study reported an AUC of 0.81 with a cut-off of 0.64 kU_A_/L for αS1-casein, 0.82 with a cut-off of 0.51 kU_A_/L for β-casein, and 0.76 with a cut-off of 0.79 kU_A_/L for κ-casein. Interestingly, for Bos d 8, the AUC was higher (0.87) compared to the individual casein fractions, with a cut-off of 0.6 kU_A_/L [10,13]. In contrast to these studies, Tosca et al. [18] reported a lower AUC of only 0.67 with a relatively high cut-off of 4.87 kU_A_/L. At a higher threshold of 2.6 kU_A_/L, Neves et al. [16] reported excellent specificity (0.95) and PPV (1.0), but moderate sensitivity (0.64) and low NPV (0.37), which limit its applicability for screening [16]. Nieminen et al. [17] proposed the highest cut-off at 16.55 kU_A_/L, achieving strong specificity (0.93) but poor sensitivity (0.11). Low sensitivity limits its practical utility [12]. The optimal cut-off values for sIgE to Bos d 8 in the enrolled studies using ImmunoCAP are shown in Figure 3. The mean cut-off value for Bos d 8 using ImmunoCAP was 4.7 kU_A_/L, and the median was 2.5 kU_A_/L with an AUC value of 0.67–0.976. Among the analyzed components, Bos d 8 showed relatively favorable diagnostic performance within the included studies, particularly with regard to AUC. These findings should be interpreted with caution, as the included studies differed substantially in terms of patient selection, age distribution, and diagnostic protocols, which limits the direct comparability of reported cut-off values and performance estimates. Although Bos d 8 appears to be a valuable marker, its diagnostic performance is context-dependent and should not be generalized across all pediatric populations, given the marked variability in cut-off values and performance estimates across different study populations and test platforms.

The cut-off values based on sIgE for Bos d 8 exhibited considerable variability across the studies analyzed. They ranged from 0 to 16.55 kU_A_/L (for ImmunoCAP, 0.35 to 16.55 kU_A_/L; for ISAC, 0 to 0.6 kU_A_/L). Sensitivity for ImmunoCAP ranged from 11% to 90%, while for ISAC, it ranged from 54% to 78%. Specificity values for ImmunoCAP testing varied between 63% and 95%, and for ISAC testing, specificity ranged from 81% to 96%. The PPV was relatively high, with a range of 0.7 to 1 for ImmunoCAP and 0.9 to 0.96 for ISAC. In contrast, the NPV for Bos d 8 sIgE was lower, ranging from 0.24 to 0.84 for ImmunoCAP and from 0.36 to 0.78 for ISAC. Further analysis of the ISAC test assessed the individual subunits of Bos d 8. This analysis reported cut-off values ranging from 0.1–0.64 for αS1, 0.1–0.54 for β, 0.2 for γ, and 0.79 for κ. Specificity was reported as high, but sensitivity was relatively low for these subunits. The AUC values ranged from 0.6 to 0.82.

### 2.5. Bos d Lactoferrin

Only the study by Petersen provided a cut-off value for Bos d lactoferrin at 0.08 kU_A_/L. However, its diagnostic utility was limited, as evidenced by an AUC of 0.58. The sensitivity was low (0.39), while the specificity was relatively high (0.86) [10].

Despite attempts to reduce differences related to nationality, geography, and cultural background, there are still large variations in the reported sIgE cut-off values for cow’s milk allergy across studies. This may be due to the lack of large, multicenter studies, as most available data come from relatively small, single-center groups, usually including no more than 170 patients [7,10,11,12,13,14,15,16,17,18].

However, a general comparative trend can be observed: older children tend to have higher sIgE levels, especially for Bos d 8. For instance, a sixfold difference in the optimal cut-off value for Bos d 8 between younger (1–2 years, cut-off: 2.61 kU_A_/L) and older children (3–14 years, cut-off: 16.55 kU_A_/L) was reported in the study by Nieminen et al. [17]. Age differences between study groups are crucial when comparing findings, as some studies include only infants, while others evaluate children across a wide age range (0–18 years) [7,16]. This matters clinically because persistent cow’s milk allergy is usually associated with higher sIgE levels and a more severe clinical course [10,12,17].

In addition to population differences, methodological discrepancies between testing methods, such as ImmunoCAP and ISAC, significantly affect the reported results. These methods differ fundamentally in how they measure and detect allergen-specific IgE. Consequently, thresholds and diagnostic performances cannot be used interchangeably between these different assay platforms [5].

To provide a comprehensive and comparative overview of the diagnostic efficacy across the analyzed studies, the ranges of sensitivity, specificity, and AUC for the main cow’s milk molecular components are summarized in Table 6. As demonstrated, Bos d 8 consistently exhibits the highest predictive value for OFC outcomes.

## 3. Discussion

Milk is rich in vital nutrients such as fats, proteins, calcium, phosphorus, and vitamin B12. It should be restricted only when necessary because it is an essential part of the child’s diet. The diagnosis of CMPA should be accurate. Clinicians should ensure that children without CMPA avoid unnecessary restrictive diets that may lead to nutritional deficiencies. It is worth emphasizing that patients on elimination diets may develop severe reactions when they accidentally ingest an allergen. The DBPCFC is the gold standard for diagnosing CMPA. This procedure carries a significant risk of anaphylactic reactions, is time-consuming and is expensive. From a clinical practice perspective, the search for alternative diagnostic methods is essential. One possible substitute is CRD, a method carried out using minimal volumes of serum obtained from capillary blood samples [7,12,13,15,16]. Optimal cut-off values for confirmed CMPA have not yet been established. The aim of our review is to clarify this issue.

CMPA is typically temporary in the majority of patients who adhere to an exclusion diet and are fed hydrolyzed or soy-based formulas. Therefore, periodic follow-up is essential to determine whether tolerance has developed. CRD offers a valuable diagnostic option in this context. One key finding from our review is that sIgE levels are influenced by the patient’s age [7,12]. Cow’s milk allergy, the most common allergy in childhood with a prevalence of 2.3%, is known to resolve in 45% to 85% of patients as they age. This explains the decreasing sIgE levels in a significant percentage of CMPA patients over time, as the allergy is outgrown. A Spanish study reported a 64% tolerance rate after three years of follow-up among CMPA patients [7]. Physicians often question whether CRD can be used to identify CMPA patients who are developing tolerance by analysing their sIgE antibody responses to CMP allergens. In 2018, Petersen et al. analyzed 30 children with persistent cow’s milk allergy and 140 tolerant children. They reported a significant increase in median casein-specific IgE levels as the patients aged. The median sIgE values started at 12.3 kU_A_/L at 24 months, peaked at 34.9 kU_A_/L by 36 months, and then slightly decreased to 30.2 kU_A_/L by 48 months. In contrast, tolerant patients had much lower median sIgE levels. These levels started at 0.34 kU_A_/L at 12 months, increased to 0.85 kU_A_/L at 18 months, and remained relatively stable at 0.66 kU_A_/L, at 24 months and 1.05 kU_A_/L at 48 months [10]. This data underscore the significant difference in sIgE levels between allergic and tolerant patients. The available literature on children developing tolerance to CM has shown that these patients tend to have lower levels of CM-specific IgE antibodies compared to those with persistent allergies [14]. Consequently, a low IgE response to Bos d 4, Bos d 5, Bos d 6, and Bos d 7 could serve as a good predictor of a negative OFC [14]. In 2018, Petersen et al. similarly observed that sIgE levels in the persistent allergy group were higher than in the tolerance groups (early and late tolerance) and the non-allergic group. They reported no significant difference in sIgE values between the early tolerance and late tolerance groups [10].

The number of patients who are either overdiagnosed or missed is a key challenge in correlating CRD to OFC outcomes [12,19]. An Italian mathematical analysis found that using the 95% confidence decision point (CDP) for CM sIgE, measured with ImmunoCAP, could eliminate 27% of OFCs. Furthermore, the sequential use of two tests could lead to a 50% reduction in OFCs, including a significant decrease in positive OFCs [13]. Italian researchers found no significant advantage in testing sIgE to individual casein fractions compared to total casein. D’Urbano et al. concluded that casein is a strong predictor of CMPA [13]. However, two independent findings showed no single milk allergen to be superior to whole cow’s milk in predicting CMPA. These conflicting findings can be largely explained by the profound clinical heterogeneity across the studied populations [10,18]. First, differences in the age of participants play a critical role; highly sensitized younger infants often display distinct immune responses compared to older children with persistent allergies, which can significantly alter the predictive value of individual components [10,12,18]. Second, a higher prevalence of comorbidities—particularly moderate-to-severe atopic dermatitis (AD) in cohorts recruited from specialized tertiary allergy centers—strongly correlates with highly elevated total and specific IgE levels [13,17]. This dynamic can artificially inflate overall cow’s milk sIgE without proportionally increasing the diagnostic accuracy of individual components like Bos d 8. Finally, variations in patient selection criteria and differing challenge protocols further contribute to these discrepancies [4,13]. It is worth noting that sensitisation to ≥5 milk proteins allergens (including casein subtypes) correlates with higher whole cow’s milk sIgE levels.

In 2020, Ayats-Vidal et al. reported a significant correlation between sIgE levels to cow’s milk and casein and the severity of allergic reactions [15]. Similar tendencies were noted in other demographic groups. For instance, among Italian children, those with sIgE levels to Bos d 8 exceeding 12.2 kU_A_/L had an odds ratio of 15 for experiencing an anaphylactic reaction compared to children with lower sIgE levels [18]. However, contradictory findings were reported by Nieminen et al. and Petersen et al., who described this association as clinically irrelevant [10,17]. Three of the enrolled studies indicate that none of the sIgE milk components can replace the DBPCFC as the gold standard [10,15,17]. On the other hand, six suggest this possibility [7,12,13,15,16].

Table 3, Table 4, Table 5 and Table 6 present the sIgE cut-off values for specific CM allergens. These values vary significantly between the reviewed papers. These differences can be attributed to multiple factors, including variations in challenge protocols, the subjective interpretation of symptoms during the OFC, and notably, the specific selection criteria of the patients. For instance, the exceptionally high cut-off value of 34.27 kU_A_/L for Bos d 4 reported by D’Urbano et al. warrants a specific explanation. Such an elevated threshold is largely driven by the underlying clinical characteristics of their cohort. Patients evaluated in specialized tertiary allergy centers frequently present with more severe allergic phenotypes, including a higher prevalence of moderate-to-severe AD. It is clinically well documented that severe AD strongly correlates with highly elevated total and specific IgE levels, which can inflate diagnostic cut-offs without necessarily indicating a higher risk of clinical reactivity to milk. Furthermore, specific age distributions—such as the inclusion of highly sensitized younger patients—can also significantly contribute to pushing these optimal diagnostic thresholds upward [17]. To tackle this challenge, a group of Italian researchers investigated whether ratios of specific IgE to total IgE could enhance the ability to predict OFC outcomes compared to specific IgE levels alone. The ratios, particularly casein-specific IgE/total IgE, showed strong predictive value for a positive OFC, but offered no significant advantage over using casein-specific IgE alone [15].

Screened articles from Europe, focusing on children aged 0–18 years and written in English, reveal significant variability in cut-off values. Thresholds ranged from low to high, and sensitivity and specificity values differed across testing methods such as ImmunoCAP and ISAC. The most comprehensive data are available for Bos d 4, Bos d 5, and Bos d 8. On the other hand, the least data exist for Bos d lactoferrin, with only one paper available, and just two studies describe Bos d 6.

Bos d 4 (α-lactalbumin) has a high PPV, making it a useful marker for diagnosing CMPA. However, its NPV for α-lactalbumin varies between 0.3 and 0.7, which emphasizes that a negative result should be interpreted with caution. A negative result may indicate the absence of the specific antigen tested or involve other immunological mechanisms.

Similarly, sIgE cut-offs for Bos d 5 (β-lactoglobulin) also have a high PPV, making it a good marker in identifying CMPA. The specificity of the tests is quite high, ranging from 71% to 93% for ImmunoCAP and 88% to 93% for ISAC. However, its sensitivity is lower (32–67% for ImmunoCAP, 50–56% for ISAC) and its NPV (ranging from 0.67 to 0.91 for ImmunoCAP and 0.7 for ISAC) suggest that a negative result should not fully exclude CMPA.

In the case of Bos d 6 (bovine albumin serum), although the PPV remains high, ranging from 0.5 to 1 for ImmunoCAP and 1 for ISAC, the low sensitivity (ranging from 6% to 8% for CAP) suggests poor utility for diagnosing CMPA and predicting OFC outcomes.

Despite variability in sensitivity, specificity, PPV, and NPV across the analyzed components, Bos d 8 showed the most favorable diagnostic performance in several studies, particularly in terms of AUC. In one study, a cut-off value of 0.95 kU_A_/L was associated with an AUC of 0.976 [15]. However, the diagnostic utility of Bos d 8 specific IgE levels must be interpreted in light of several important limitations. First, the proposed cut-off values vary significantly across studies, ranging from 0.35 to 16.55 kU_A_/L [7,10,11,12,13,14,15,16,17,18]. This discrepancy is largely driven by the fact that diagnostic accuracy and optimal thresholds differ significantly depending on the age group [10,17,18]. For instance, Nieminen et al. demonstrated that older children require a much higher cut-off value to predict a positive OFC compared to toddlers, highlighting the need for age-adjusted interpretations [17]. Furthermore, while Bos d 8 demonstrates a high PPV in multiple cohorts, its sensitivity and NPV remain largely insufficient to safely replace the OFC [12,13,16,17]. Because a low NPV implies that a negative test result cannot definitively rule out clinical reactivity, the OFC remains the irreplaceable gold standard for diagnosing CMPA [1,2,4]. Therefore, component-resolved diagnostics involving Bos d 8 should not be viewed as an absolute substitute for OFCs. Instead, it should be emphasized that Bos d 8 is a highly valuable adjunctive tool for risk stratification, aiding clinicians in avoiding potentially dangerous and time-consuming provocations in patients with the highest risk of severe reactions.

To translate these findings into daily clinical practice, the practical implications of sIgE thresholds must be clearly outlined. First, clinicians should utilize sIgE cut-offs, particularly for Bos d 8, primarily as a tool for risk stratification rather than a definitive diagnostic test. For instance, patients with sIgE levels exceeding identified high-risk thresholds (such as 12.2 kU_A_/L for Bos d 8) have a significantly higher odds ratio for experiencing severe anaphylactic reactions [12]. In such high-risk cases, CRD can guide the clinician to postpone the OFC, thereby maximizing patient safety. Second, while CRD cannot completely replace the OFC due to low negative predictive values [12,13,16,17], using statistically derived decision points can substantially reduce the number of unnecessary procedures. As demonstrated by D’Urbano et al., applying a 95% CDP for cow’s milk sIgE could eliminate 27% of OFCs, and a sequential testing approach could reduce them by up to 50% [13]. Finally, CRD has profound clinical implications for monitoring the development of tolerance. Sequential measurements of sIgE, especially against casein, can effectively differentiate between persistent allergy and resolving CMPA. Children outgrowing their allergy typically exhibit a gradual decline in sIgE levels, whereas those with persistent CMPA show persistently high or increasing allergen-specific IgE concentrations [10,17]. Consequently, a steady decrease in Bos d 8 sIgE can signal to the clinician the optimal and safest timing to perform a confirmatory OFC, whereas rising levels strongly suggest the need to maintain the elimination diet [10,14]. Importantly, the clinical utility of CRD should always be interpreted in conjunction with detailed patient history and clinical presentation, rather than used as a standalone diagnostic tool.

Specific IgE tests for these cow’s milk proteins show promise in diagnosing CMPA. The clinical interpretation must consider both the strengths and limitations of these tests. High PPV values indicate that positive results are generally reliable, but the low NPV values for most molecules suggest that negative results should not be used to rule out allergy, especially in cases where the clinical manifestation strongly suggests milk allergy. To conclude, in the screened articles, the lowest AUC value was observed for Bos d 6 and Bos d lactoferrin [10,17]. Among the reviewed papers, Bos d 8 demonstrated the highest AUC value, with an AUC of 0.976 at a cut-off of 0.95 kU_A_/L in ImmunoCAP and making Bos d 8 a highly valuable indicator for assessing the risk of a positive OFC outcome [15]. There are several possible biases in this study. The potential limitations of this review are that we included studies originating from Europe and involving children aged 0–18 years, which may exclude relevant research from other regions or age groups. In addition, restricting the review to English-language publications may not comprehensively represent all important studies published in other languages. Furthermore, relying solely on specific databases may overlook relevant studies that are available from other sources.

### Limitations

A notable limitation of this review is its geographical restriction. By focusing exclusively on European studies, the generalizability of our findings to global pediatric populations is reduced. Because the proposed sIgE cut-off values and sensitization profiles are highly population-dependent and vary across regions, the diagnostic thresholds identified in this review should be applied with caution in non-European clinical settings. Future global studies or region-specific reviews (e.g., focusing on Asian or American cohorts) are needed to establish localized predictive cut-off values for CMPA.

To fully contextualize our findings, it is crucial to address the methodological quality and inherent limitations of the primary studies included in this review. A structured evaluation reveals several sources of potential bias that must be considered when interpreting the reported cut-off values. Due to the narrative design of this review, a formal risk of bias assessment using standardized tools (e.g., QUADAS-2) was not performed; instead, potential sources of bias were evaluated qualitatively. First, regarding study design and selection bias, exactly half of the analyzed studies (5 out of 10) were retrospective in design [10,15,16,18], which intrinsically carries a risk of selection and information bias due to reliance on historical medical records. Additionally, because most of these studies were conducted in specialized tertiary allergy or pediatric centres, the cohorts likely represent patients with more severe allergic phenotypes rather than the general population, thereby introducing referral bias. Another major limitation is age heterogeneity. The mean age of participants varied drastically across the studies, ranging from young infants (e.g., a mean of 4.8 months) to older children and adolescents up to 18 years old [7,16]. Because sIgE levels, immune responses, and the likelihood of developing tolerance are highly age-dependent [10,17,18], this immense variability complicates the direct comparison of optimal cut-offs across different cohorts. Finally, there is significant variability in OFC protocols and CMPA definitions. A notable lack of standardization was observed in the challenge protocols and the clinical criteria used to define a ‘positive’ CMPA outcome. While the DBPCFC remains the strict gold standard [1,2,4], many of the included studies relied on open food challenges for ethical and practical reasons. The subjective interpretation of mild or delayed symptoms during these non-blinded challenges may have introduced misclassification bias. Acknowledging these limitations highlights the urgent need for large, prospective, multicentre studies using standardized DBPCFC protocols to validate universally applicable CRD thresholds. This study provides a clinically relevant synthesis of the currently available evidence, supporting a more individualized and risk-adapted approach to CMPA diagnostics in clinical practice.

## 4. Materials and Methods

**Figure 4:**
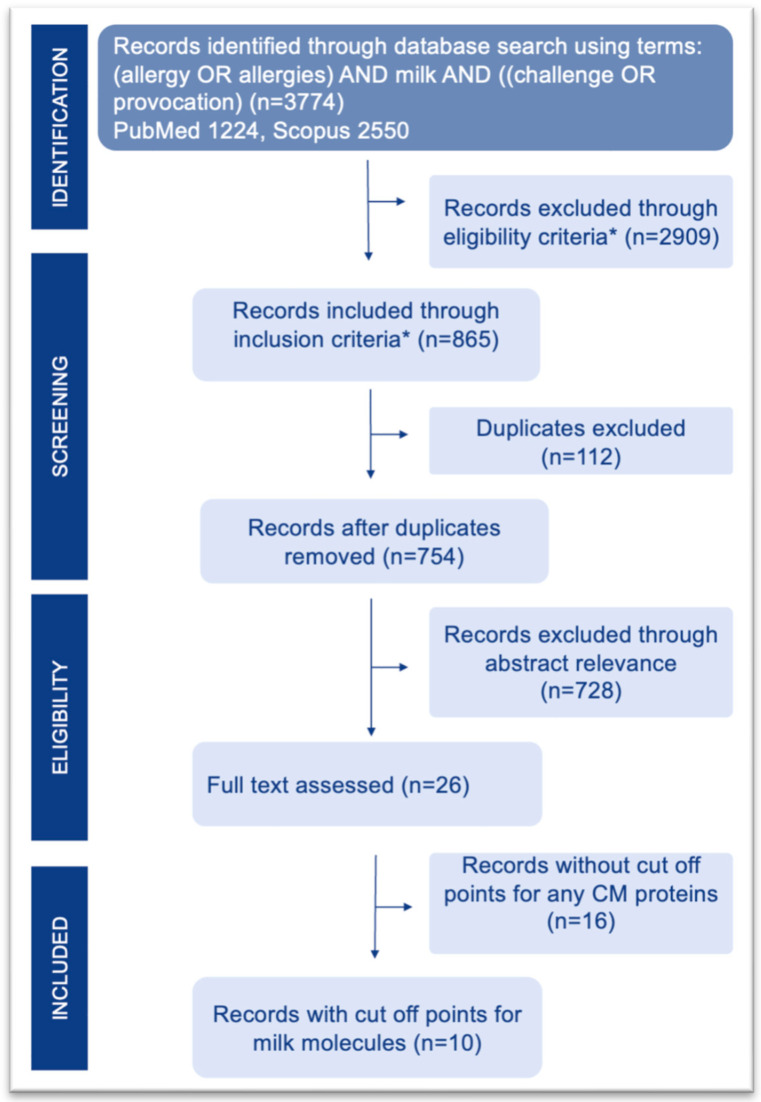
Flow chart of the search process. * inclusion and exclusion criteria were applied as described in the Materials and Methods.

This article was designed as a narrative review. A formal quantitative meta-analysis was not feasible due to the substantial methodological heterogeneity across the included primary studies. Specifically, the use of non-comparable sIgE cut-off thresholds, the frequent lack of reported standard errors for predictive estimates, and the widely varying clinical criteria used to define a positive OFC outcome precluded the statistical pooling of the data. Consequently, the data synthesis presented in this study is strictly qualitative and narrative. Therefore, any attempt at quantitative synthesis could have produced misleading pooled estimates and was deliberately avoided. However, to ensure transparency, minimize selection bias, and provide a comprehensive overview of the literature, a structured search strategy was applied. A search was conducted independently in 2024 by two independent pairs of authors in parallel, using PubMed and Scopus, with the search terms (‘allergy’ OR ‘allergies’) AND ‘milk’ AND (‘challenge’ OR ‘provocation’). In total, a search using the keywords in the titles and abstracts across two databases returned 3774 publications. Of these, 2909 articles were excluded based on eligibility criteria. Duplicates (n = 112) were then removed. In the next step, abstracts were manually screened for relevance, resulting in 26 articles being selected. Full texts were assessed to select studies containing data on molecular thresholds for the prediction of OFC outcomes on milk protein sensitization. The final number of studies included in the analysis was 10 (Figure 4).

## 5. Conclusions

Our review of component-resolved diagnostics in pediatric CMPA underscores the crucial role of Bos d 8. Among all evaluated milk proteins, Bos d 8 consistently demonstrated the highest diagnostic accuracy, peaking at an AUC value of 0.976 at a cut-off of 0.95 kU_A_/L in ImmunoCAP in one study, making it the most promising molecular indicator for assessing the risk of positive OFC outcomes. Nevertheless, this conclusion must be moderated by the substantial clinical heterogeneity across the reviewed literature, as some research indicates that no single milk component significantly outperforms whole cow’s milk specific IgE. However, its interpretation requires caution, as sIgE levels primarily reflect sensitization and the high variability of proposed cut-offs and low NPVs across different populations, Bos d 8 cannot be considered an absolute predictor of clinical reactivity. While the DBPCFC remains the irreplaceable gold standard for diagnosing CMPA, the integration of Bos d 8 into daily clinical practice provides a valuable tool for risk stratification, potentially reducing the reliance on risky and time-consuming OFCs in strongly sensitized individuals.

## Figures and Tables

**Table 1:** Summary of original studies assessing the impact of component-resolved diagnostics on OFC.

No.	Study	No. All Patients/with CMPA	Age Cohort (Mean Age)	Region /Type of Study	Tests Used For Diagnosis of Allergy	Tested Allergens	The Cut-Off Points of sIgE Values Used to Recommend Whether to Proceed OFC or Not	% Positive CM OFC/No. CM OFC/ Protocol Type
1	García-Ara et al. 2001 [7]	170/76	1–12 months/ (4.8 months)	Spain/prospective	SPT Alergia e Inmunología Abelló (Madrid, Spain)/Sigma Chemical (St. Louis, MO, USA)	Bos d 4 Bos d 5 Bos d 8		42%/161 Open OFC
					sIgE ImmunoCAP system FEIA (Pharmacia Diagnostics, Uppsala, Sweden)	Bos d 4	0.35 kU_A_/L	
						Bos d 5	0.35 kU_A_/L	
						Bos d 8	0.35 kU_A_/L	
2	Ott et al. 2008 [11]	130/85	5–150 months (14 months)	Germany/retrospective	SPT	fresh cow’s milk 3.5% fat		49%/85 Mixed
					sIgE UniCAP^TM^, Phadia, Uppsala, Sweden/ISAC^TM^; VBC Genomics Bioscience Research, Vienna, Austria	Bos d 4	0.1 kU_A_/L	
						Bos d 5	0.1 kU_A_/L	
						α-casein	0.1 kU_A_/L	
						β-casein	0.1 kU_A_/L	
						γ-casein	0.2 kU_A_/L	
3	Martorell et al. 2008 [12]	170/170	1–12 y (5.4 months)	Spain/ prospective	SPT (Laboratorios Leti CBF, Barcelona, Spain)	Bos d 4 Bos d 5 Bos d 8		Open OFC
					sIgE ImmunoCAP system FEIA (Pharmacia Diagnostics, Uppsala, Sweden)	Bos d 8 12 mth	0.97 kU_A_/L	66%/407
						Bos d 8 18 mth	1.22 kU_A_/L	45%/407
						Bos d 8 24 mth	3 kU_A_/L	31%/407
						Bos d 8 36 mth	2.39 kU_A_/L	21%/407
						Bos d 8 48 mth	2.73 kU_A_/L	18%/407
4	D’Urbano et al. 2010 [13]	104/32	0.7–15.1 y (4.9 y)	Italy/ prospective	SPT (Lofarma, Milan, Italy)	Bos d 4 Bos d 5 Bos d 8		55%/58 Open OFC
					sIgE ImmunoCAP SystemTM, Phadia Diagnostics, Uppsala, Sweden	Bos d 4	34.27 kU_A_/L	
						Bos d 5	9.91 kU_A_/L	
						Bos d 8	0.78 kU_A_/L	
					sIgE (ISAC^TM^ version CRD89)	Bos d 4	2.27 ISU	
						Bos d 6	44.32 ISU	
						Bos d 8	0.6 ISU	
						αS1-casein	0.64 ISU	
						β-casein	0.51 ISU	
						κ-casein	0.79 ISU	
5	Alessandri et al. 2012 [14]	75/54	0.5–16 y (3 y)	Italy/ prospective	SPT Lofarma, Milan, Italy	Bos d 4 Bos d 5 Bos d 8		77%/70 Mixed
					sIgE ImmunoCAP system (Phadia, Sweden)	Bos d 4	1.02 kU_A_/L	
						Bos d 5	0 kU_A_/L	
						Bos d 8	0.44 kU_A_/L	
					ISAC 103 (Phadia Multiplexing Diagnostics, PMD, Vienna, Austria)	Bos d 4	0 ISU	
						Bos d 5	0 ISU	
						Bos d 8	0 ISU	
6	Petersen et al. 2018 [10]	78/39	6–204 months (3.5 y)	Denmark/retrospective	SPT Soluprick^®^ ALK-ABELLÓ, Hørsholm, Denmark	Pasteurized low-fat milk		50%/78 Open OFC
					sIgE ImmunoCAP (Thermo Fisher Scientific, Uppsala, Sweden)	Bos d 4	0.77 kU_A_/L	
						Bos d 5	1.59 kU_A_/L	
						Bos d 8	≥2.33 kU_A_/L	
						Bos d lactoferrin	0.08 kU_A_/L	
7	Ayats-Vidal et al. 2020 [15]	138/39	0.75–15 y (4 y)	Spain/retrospective	SPT	Bos d 4 Bos d 5 Bos d 8		54%/72 Open OFC
					sIgE ImmunoCAP^®^, (TermoFisher Scientific, Uppsala, Sweden)	Bos d 4	0.64 kU_A_/L	
						Bos d 5	0.61 kU_A_/L	
						Bos d 8	0.64 kU_A_/L	
8	Castro Neves et al. 2020 [16]	105/83	0–18y (2.5 y)	Portugal/ retrospective	SPT Bial Aristegui^®^, ALK-Abelló^®^	Bos d 4 Bos d 5 Bos d 8		100%/37 Open OFC
					sIgE ImmunoCAP^®^ method (Thermo Fisher Scientific^®^, Uppsala, Sweden)	Bos d 4	1.6 kU_A_/L	
						Bos d 5	1.7 kU_A_/L	
						Bos d 8	2.6 kU_A_/L	
9	Otso Nieminen et al. 2023 [17]	130/98	1–14.1 y (1.8 y)	Finland/prospective	sIgE ImmunoCAP^®^, (ThermoFisher Scientific, Uppsala, Sweden)	Bos d 4	6.25 kU_A_/L	75%/130 Open OFC
						Bos d 5	1.94 kU_A_/L	
						Bos d 6	0.49 kU_A_/L	
						Bos d 8	16.55 kU_A_/L	
10	Tosca et al. 2023 [18]	72/30	(71.6 months)	Italy/retrospective	sIgE ELISA assays TermoFisher (Milan, Italy)	Bos d 4	1.47 kU_A_/L	41.6%/72 Open OFC
						Bos d 5	1.35 kU_A_/L	
						Bos d 8	4.87 kU_A_/L	

CM—cow’s milk; CMPA—cow’s milk protein allergy; OFC—oral food challenge; SPT—skin prick test; sIgE—specific immunoglobulin E.

**Table 2:** Summary of original studies evaluating the impact of sIgE to Bos d 4 on oral food challenge.

Method (Unit)	Study	No. All Patients/with CMPA	Cut-Off “+” OFC	Sensitivity	Specificity	PPV	NPV	AUC	% of “+” CM OFC /No of CM OFC
ImmunoCAP (kU_A_/L)	García-Ara et al. 2001 [7]	170/76	0.35	0.55	0.84	0.74	0.7	N/R	42%/161
	D’Urbano et al. 2010 [13]	104/32	34.27	N/R	N/R	1	0.48	0.77	55%/58
	Alessandri et al. 2012 [14]	70/54	1.02	0.58	0.81	0.91	0.38	N/R	77%/70
	Petersen et al. 2018 [10]	78/39	0.77	0.53	0.81	N/R	N/R	0.67	50%/78
	Ayats-Vidal et al. 2020 [15]	138/39	2.25	0.67	0.91	N/R	N/R	0.908	54%/72
	Castro Neves et al. 2020 [16]	105/83	1.6	0.55	0.86	1	0.52	N/R	100%/37
	Nieminen et al. 2023 (1–2 yo) [17]	96/N/R	0.76	0.52	0.93	0.97	0.3	0.79	75%/130
	Nieminen et al. 2023 (3–14 yo) [17]	34/N/R	6.25	0.32	0.92	0.83	0.52	0.714	
	Tosca et al. 2023 [18]	72/30	1.47	0.62	0.71	N/R	N/R	0.68	41.6%/72
ISAC (ISU)	Ott et al. 2008 [11]	130/85	0.1	0.5	0.93	0.875	0.656	0.7	49%/85
	D’Urbano et al. 2010 [13]	104/32	2.27	N/R	N/R	1	0.51	0.7	55%/58
	Alessandri et al. 2012 [14]	70/54	0	0.56	0.88	0.93	0.39	N/R	77%/70

CMPA—cow’s milk protein allergy; OFC—oral food challenge; PPV—positive predictive value; NPV—negative predictive value; AUC—the area under the ROC curve; CM—cow’s milk; N/R—not reported.

**Table 3:** Summary of original studies evaluating the impact of sIgE to Bos d 5 on oral food challenge.

Method (Unit)	Study	No. All Patients /with CMPA	Cut-Off “+” OFC [kU_A_/L]	Sensitivity	Specificity	PPV	NPV	AUC	% Positive CM OFC/No. CM OFC
ImmunoCAP (kU_A_/L)	García-Ara et al. 2001 [7]	170/76	0.35	0.59	0.8	0.7	0.71	N/R	42%/161
	Alessandri et al. 2012 [14]	70/54	0.9	0.5	0.85	0.62	N/R		77%/70
	Petersen et al. 2018 [10]	78/39	1.59	0.44	0.84	N/R	N/R	0.67	50%/78
	Ayats-Vidal et al. 2020 [15]	138/39	1.6	0.82	0.91	N/R	N/R	0.92	54%/72
	Castro Neves et al. 2020 [16]	105/83	1.7	0.58	0.95	1	0.44	N/R	100%/37
	Nieminen et al. 2023 (3–14 yo) [17]	34/	1.94	0.38	0.92	0.86	0.55	0.74	75%/130
	Nieminen et al. 2023 (1–2 yo) [17]	96/	3.14	0.3	0.93	0.95	0.22	0.73	
	Tosca et al. 2023 [18]	72/30	1.35	0.59	0.68	N/R	N/R	0.68	41.6%/72
	D’Urbano et al. 2010 [13]	104/32	9.91	N/R	N/R	1	0.5	0.72	55%/58
ISAC (ISU)	Ott et al. 2008 [11]	130/85	0.1	0.24	0.95	0.83	0.56	0.6	49%/85
	Alessandri et al. 2012 [14]	70/54	0	0.4	0.94	0.95	0.33	N/R	77%/70

CMPA—cow’s milk protein allergy; OFC—oral food challenge PPV—positive predictive value; NPV—negative predictive value; AUC—the area under the ROC curve; CM—cow’s milk; N/R—not reported.

**Table 4:** Summary of original studies evaluating the impact of sIgE to Bos d 6 on oral food challenge.

Method (Unit)	Study	No. All Patients/with CMPA	Cut-Off “+” OFC [kU_A_/L]	Sensitivity	Specificity	PPV	NPV	AUC	% Positive CM OFC/No. CM OFC
ImmunoCAP (kU_A_/L)	Nieminen et al. 2023 (1–2 yo) [17]	96/	0.26	0.08	0.93	0.86	0.18	0.58	75%/130
	Nieminen et al. 2023 (3–14 yo) [17]	34/	0.49	0.06	0.92	0.5	0.44	0.413	
	D’Urbano et al. 2010 [13]	104/32	9.91	N/R	N/R	1	0.5	0.72	55%/58
ISAC (ISU)	D’Urbano et al. 2010 [13]	104/32	44.32	N/R	N/R	1	0.46	0.59	55%/58

CMPA—cow’s milk protein allergy; OFC—oral food challenge; PPV—positive predictive value; NPV—negative predictive value; AUC—the area under the ROC curve; CM—cow’s milk; N/R—not reported.

**Table 5:** Summary of original studies evaluating the impact of sIgE to Bos d 8 on oral food challenge.

Method (Unit)	Study	No. All Patients /with CMPA	Casein Types	Cut-Off “+” OFC [kU_A_/L]	Sensitivity	Specificity	PPV	NPV	AUC	% Positive CM OFC/No. CM OFC
ImmunoCAP (kU_A_/L)	García-Ara et al. 2001 [7]	170/76	Bos d 8	0.35	0.71	0.75	0.7	0.76	N/R	42%/161
	D’Urbano et al. 2010 [13]	104/32	Bos d 8	0.78	N/R	N/R	0.85	0.84	0.93	55%/58
	Alessandri et al. 2012 [14]	70/54	Bos d 8	0.44	0.82	0.63	0.87	0.53		77%/70
	Petersen et al. 2018 [10]	78/39	Bos d 8	2.33	0.61	0.83	N/R	N/R	0.77	50%/78
	Ayats-Vidal et al. 2020 [15]	138/39	Bos d 8	0.95	0.89	0.91	N/R	N/R	0.976	54%/72
	Castro Neves et al. 2020 [16]	105/83	Bos d 8	2.6	0.64	0.95	1	0.37	N/R	100%/37
	Nieminen et al. 2023 (1–2 yo) [17]	96/	Bos d 8	2.61	0.34	0.94	0.96	0.24	0.77	75%/130
	Nieminen et al. 2023 (3–14 yo) [17]	34/	Bos d 8	16.55	0.11	0.93	0.68	0.43	0.697	
	Tosca et al. 2023 [18]	72/30	Bos d 8	4.87	0.52	0.82	N/R	N/R	0.67	41.6%/72
	Martorell et al. 2008 (12 mth) [12]	170/170	Bos d 8	0.97	0.68	0.81	0.8	0.7	N/R	66%
	Martorell et al. 2008 (18 mth) [12]		Bos d 8	1.22	0.7	0.63	0.8	0.51	N/R	45%
	Martorell et al. 2008 (24 mth) [12]		Bos d 8	3	0.79	0.8	0.9	0.62	N/R	31%
	Martorell et al. 2008 (36 mth) [12]		Bos d 8	2.39	0.9	0.78	0.9	0.78	N/R	21%
	Martorell et al. 2008 (48 mth) [12]		Bos d 8	2.73	0.85	0.83	0.95	0.55	N/R	18%
ISAC (ISU)	D’Urbano et al. 2010 [13]	104/32	Bos d 8	0.6	0.78	0.96	0.96	0.78	0.87	55%/58
			αS1-casein	0.64	N/R	N/R	0.95	0.64	0.81	
			β-casein	0.51	N/R	N/R	1	0.67	0.82	
			κ-casein	0.79	N/R	N/R	1	0.6	0.76	
	Alessandri et al. 2012 [14]	70/54	Bos d 8	0	0.54	0.81	0.9	0.36	N/R	77%/70
	Ott et al. 2008 [11]	130/85	αS1-casein	0.1	0.26	0.98	0.92	0.58	0.6	49%/85
			β-casein	0.1	0.26	0.93	0.79	0.56	0.6	
			κ-casein	0.2	0.38	0.88	0.76	0.59	0.6	

CMPA—cow’s milk protein allergy; OFC—oral food challenge; PPV—positive predictive value; NPV—negative predictive value; AUC—the area under the ROC curve; CM—cow’s milk; N/R—not reported.

**Table 6:** Summary of diagnostic performance of milk components.

Component	Sensitivity	Specificity	AUC	Key Observation
Bos d 8	11–90%	63–95%	0.67–0.976	highest predictive value
Bos d 4	32–67%	71–93%	0.67–0.908	variable performance
Bos d 5	30–82%	68–95%	0.6–0.92	moderate utility
Bos d 6	6–8%	92–93%	0.41–0.72	low sensitivity

## Data Availability

No new primary data were generated in this narrative review. All data supporting the findings of this study were extracted from previously published articles identified through the structured literature search described in the Section 4. The extracted dataset (screening log and study-level extraction sheet) is available from the corresponding author on reasonable request.
